# Clinical and Epidemiological Characteristics of Retrospective Single-Center Observational Study

**DOI:** 10.3390/jcm15186960

**Published:** 2026-09-08

**Authors:** Elena Cristina Andrei, Ilona Mihaela Liliac, Gabriela Boldeanu, Cristina Jana Busuioc, Larisa Pătru, Mihaela Jana Țuculina, Oprea Valentin Bușu, Cătălina Alexandra Iacov, Ciprian Laurențiu Pătru, Cristina Maria Munteanu

**Affiliations:** 1 Department of Histology, Faculty of Dentistry, University of Medicine and Pharmacy of Craiova, 2 Petru Rareș Street, 200349 Craiova, Romania; elena.andrei@umfcv.ro; 2 Department of Histology, Faculty of Medicine, University of Medicine and Pharmacy of Craiova, 2 Petru Rareș Street, 200349 Craiova, Romania; cristina.busuioc@umfcv.ro; 3 Doctoral School, University of Medicine and Pharmacy of Craiova, 2 Petru Rareș Street, 200349 Craiova, Romania; gabrielaboldeanu@gmail.com (G.B.); catalina.iacov0798@gmail.com (C.A.I.); 4 Department 9, Faculty of Medicine, University of Medicine and Pharmacy of Craiova, 2 Petru Rareș Street, 200349 Craiova, Romania; 5 Department of Endodontics, Faculty of Dentistry, University of Medicine and Pharmacy of Craiova, 2 Petru Rareș Street, 200349 Craiova, Romania; 6 Department of Educational Sciences and Communication, Faculty of Letters, University of Craiova, 13 A.I. Cuza Street, 200585 Craiova, Romania; valentin_busu@yahoo.com; 7 Department of Obstetrics and Gynecology, Faculty of Medicine, University of Medicine and Pharmacy of Craiova, 2 Petru Rareș Street, 200349 Craiova, Romania; patru.ciprianl@gmail.com; 8 Department of Oral and Maxillofacial Surgery, Faculty of Dentistry, University of Medicine and Pharmacy of Craiova, 2 Petru Rareș Street, 200349 Craiova, Romania; cristina.munteanu@umfcv.ro

**Keywords:** bisphosphonate-related osteonecrosis of the jaw (BRONJ), bisphosphonates, osteoporosis, malignant diseases, oral and maxillofacial surgery

## Abstract

**Background**: Bisphosphonate-related osteonecrosis of the jaw (BRONJ) is a severe adverse event associated with antiresorptive therapy, particularly bisphosphonates, and represents a significant challenge in oral and maxillofacial practice. This study aimed to characterize the epidemiological profile, clinical presentation, and anatomical distribution of bisphosphonate-related osteonecrosis of the jaw (BRONJ) and to investigate potential associations between demographic variables and major underlying systemic diseases. **Methods**: A retrospective single-center observational study was performed using medical records collected between January 2020 and December 2025 at the Emergency County Clinical Hospital of Craiova. After data validation and removal of duplicate records, 121 patients diagnosed with osteonecrosis of the jaw were identified. Among them, 94 patients fulfilled the inclusion criteria for BRONJ and were included in both the statistical and clinical analyses. Demographic characteristics, systemic diseases, bisphosphonate therapy, lesion localization, clinical manifestations, and statistical associations were evaluated using appropriate non-parametric and categorical statistical methods. **Results**: The final analytical sample comprised 94 patients with BRONJ associated with bisphosphonate therapy. Of these, 75 (79.8%) had received bisphosphonates for malignant disease and 19 (20.2%) for osteoporosis. The mandible was affected in 77% of patients, predominantly in the posterior premolar–molar region, whereas maxillary lesions mainly involved the posterior maxilla and maxillary tuberosity. Exposed necrotic bone was identified in all patients, while purulent discharge and oral fistulas were recorded in 37% and 35% of cases, respectively. Associations involving sex, age, residence, diabetes, hypertension, and other comorbidities were re-evaluated using the final 94-patient cohort. **Conclusions**: Among the BRONJ cases identified at this referral center, most patients had received bisphosphonate therapy for malignant disease, and lesions showed a marked predilection for the mandible, particularly its posterior region. These findings describe the clinical profile of the cases managed at our institution but do not permit estimation of BRONJ incidence or comparative risk between treatment regimens. The results support careful dental assessment before bisphosphonate therapy and multidisciplinary follow-up of patients receiving antiresorptive treatment.

## 1. Introduction

Bisphosphonates, a class of drugs widely used to treat bone-related conditions, function by increasing bone mass, boosting bone density, and reducing osteoclast activity and bone resorption. Biologically, bisphosphonates are made up of two phosphonate groups connected by a carbon atom, giving them resistance to breakdown in acidic conditions or by pyrophosphatases [1].

These drugs can be taken orally or intravenously. Injectable bisphosphonates are more effective and are used for illnesses such as Paget’s disease, bone metastases, multiple myeloma, and malignant hypercalcemia, but oral bisphosphonates are often used to treat osteoporosis, especially in elderly people [2,3]. Bisphosphonates may cause osteonecrosis of the jaw following both oral and intravenous therapy [4]. The frequent administration of bisphosphonates has additional negative side effects, including fever, sore throat, and ulcer development, which reduce the adherence of the treatment [5,6].

Bisphosphonate-related osteonecrosis of the jaw (BRONJ) is a rare occurrence but a potentially serious side effect associated mostly with antiresorptive medications such as bisphosphonates and denosumab, as well as some antiangiogenic and immune-modulating therapies.

The American Association of Oral and Maxillofacial Surgeons’ 2022 position paper defines BRONJ as the concurrent presence of three criteria: ongoing or prior treatment with antiresorptive therapy, either alone or in association with immune modulators or antiangiogenic agents; bone exposure, or bone accessible through an intraoral or extraoral fistula in the maxillofacial region, persisting for more than eight weeks; and no previous radiotherapy to the jaws or evidence of metastatic disease involving the jaws. BRONJ has replaced the term bisphosphonate-related osteonecrosis of the jaw because the condition may be associated with several pharmacological classes [7,8]. Invasive dentoalveolar procedures, especially tooth extraction, are among the most frequently reported precipitating events in patients exposed to bisphosphonates, though BRONJ may also develop spontaneously.

Surgical disruption of the oral mucosa and alveolar bone may hinder local healing in the context of impaired bone remodeling, allowing infection and inflammation to persist and eventually lead to the formation of exposed necrotic bone. However, the severity of this risk varies depending on the underlying condition, the dosage and the mode of administration of the antiresorptive medication, and cumulative therapy exposure. The risk is significantly higher among individuals with malignant conditions receiving high-dose intravenous medication, although BRONJ remains uncommon in people treated with oral bisphosphonates for osteoporosis [9,10,11].

Furthermore, periodontal and periapical inflammatory conditions may contribute substantially to BRONJ onset, suggesting that tooth extraction should be considered as part of a complex pathogenic process rather than a single causal event [12].

Although BRONJ is well recognized, its clinical presentation varies according to patient characteristics, the underlying indication for antiresorptive treatment, and the anatomical site involved. In routine referral practice, detailed descriptions integrating demographic characteristics, systemic disease, lesion distribution, and clinical stage within the same well-defined population remain useful for identifying local patterns of presentation and for improving clinical surveillance. Importantly, case-only studies cannot estimate treatment-related incidence or compare the risk associated with different bisphosphonate regimens because the total exposed population is not available. Therefore, the aim of the present study was to characterize the demographic, systemic, anatomical, and clinical features of patients with BRONJ associated with bisphosphonate therapy treated at a single tertiary referral center over a six-year period and to explore associations among selected patient characteristics, underlying diseases, and clinical stage.

## 2. Material and Methods

### 2.1. Study Design

**Figure 1:**
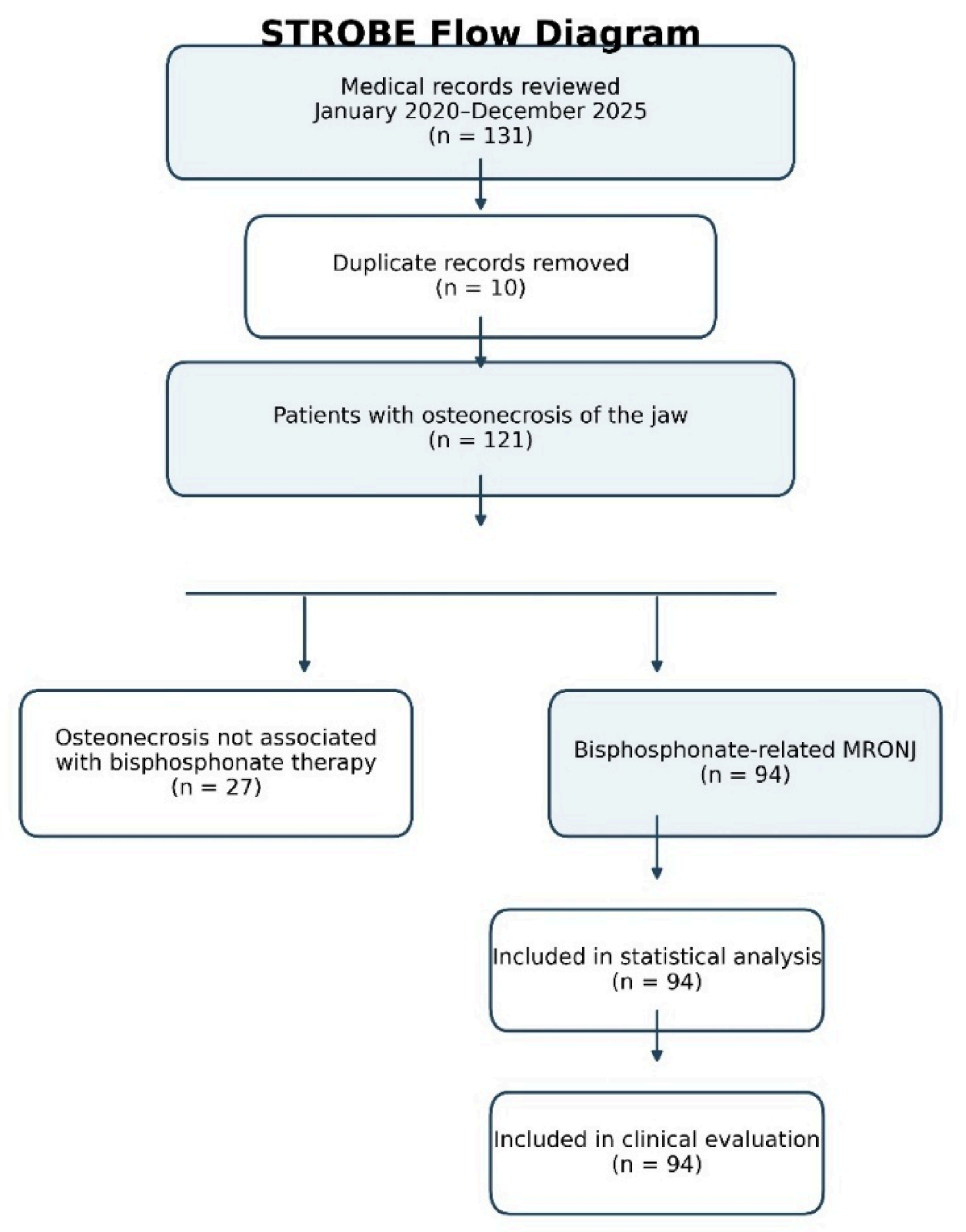
STROBE Flow Diagram.

**Figure 2:**
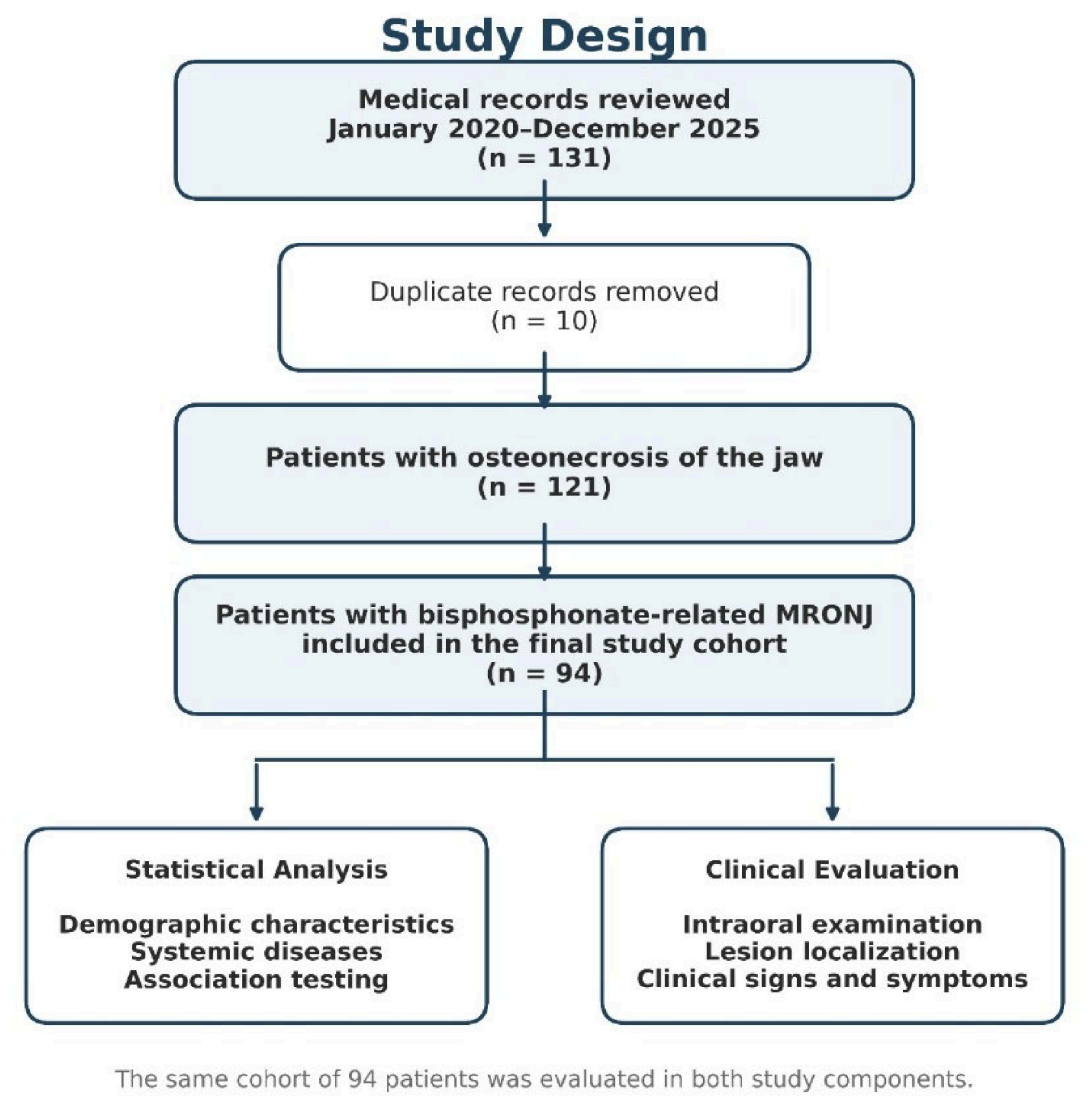
Study design.

The present investigation was designed as a retrospective, single-center observational study based on all eligible cases managed at the Oral and Maxillofacial Surgery Department of the Emergency County Clinical Hospital of Craiova between January 2020 and December 2025. The initial dataset contained 131 medical records. After removal and consolidation of 10 duplicate records, 121 unique patients with osteonecrosis of the jaw remained. Of these, 27 cases were not associated with bisphosphonate therapy and were excluded from the disease-specific analyses. The final analytical sample therefore comprised 94 patients with BRONJ associated with bisphosphonate therapy (Figure 1). The same 94 patients were used for both the statistical analyses and the detailed clinical evaluation. Because the study included BRONJ cases only and did not include a non-BRONJ comparison group or the total population exposed to bisphosphonates, the study was designed to describe case characteristics and exploratory associations rather than incidence, treatment-related risk, or causal effects (Figure 2).

### 2.2. Statistical Analysis

Statistical analyses were restricted to the final cohort of 94 patients with osteonecrosis of the jaw associated with bisphosphonate therapy. The source database contained 131 records collected between January 2020 and December 2025. Duplicate entries were identified by concordance of normalized patient name, sex, and age and were consolidated while retaining relevant clinical information. This procedure yielded 121 unique patients with osteonecrosis of the jaw; 27 patients whose osteonecrosis was not associated with bisphosphonate exposure were subsequently excluded from the BRONJ-specific analyses. Accordingly, all descriptive and inferential analyses reported for the study population should use *N* = 94 as the analytical denominator.

Categorical variables were summarized as absolute frequencies and percentages, together with Wilson 95% confidence intervals for the principal proportions. Continuous variables were described using the mean ± standard deviation, median, and interquartile range. The distribution of age was assessed with the Shapiro–Wilk test. As age did not follow a normal distribution, comparisons between patient groups were performed using the two-sided Mann–Whitney U test, and the rank–biserial correlation coefficient was reported as a measure of effect size.

Associations between binary categorical variables were evaluated using the two-sided Fisher’s exact test. Odds ratios together with their corresponding 95% confidence intervals were calculated using the Woolf logarithmic method, applying the Haldane–Anscombe correction whenever zero frequencies occurred within contingency tables. Relationships involving age categories were examined using Pearson’s chi-square test, whereas Monte Carlo *p*-values based on 50,000 random permutations were estimated when the assumptions of the chi-square test were not fully satisfied because of low expected cell counts. Effect sizes were expressed as Phi and Cramér’s V coefficients. All statistical analyses were two-sided, statistical significance was established at *p* < 0.05, and no correction for multiple testing was applied because the analyses were considered exploratory.

No a priori sample-size calculation was performed because this was a retrospective study and all eligible BRONJ cases available during the predefined six-year study period were included. Thus, the sample size was determined by case availability rather than by prospective recruitment targets. The final sample of 94 patients was considered adequate for descriptive characterization of the institutional case series; however, subgroup comparisons, particularly those involving small cell counts, may have limited statistical power and should be interpreted as exploratory.

During the preparation of this manuscript, ChatGPT (GPT-5.6 Sol, OpenAI, San Francisco, CA, USA) was used as an artificial intelligence-assisted tool for language editing, grammatical correction, and improvement of the clarity and readability of the text.

### 2.3. Clinical Study

The clinical evaluation involved the same 94 patients included in the final analytical sample. All patients had BRONJ and had been managed at the Emergency County Clinical Hospital of Craiova between January 2020 and December 2025. Bisphosphonate therapy had been prescribed for malignant disease in 75 patients and for osteoporosis in 19. Clinical examination assessed exposed necrotic bone, bone discoloration, halitosis, fistula formation, purulent discharge, lesion location, and patient-reported symptoms. Mandibular involvement was recorded in 77% of cases and maxillary involvement in 23%, with no simultaneous involvement of both jaws. Posterior sites predominated in both the mandible and maxilla. These findings describe the distribution of BRONJ cases within the study sample and should not be interpreted as estimates of treatment-specific risk.

### 2.4. Inclusion Criteria

Patients were eligible for inclusion if they fulfilled all of the following criteria:

(i) A confirmed diagnosis of bisphosphonate-related osteonecrosis of the jaw (BRONJ) associated with bisphosphonate therapy, established by clinical examination in accordance with current diagnostic recommendations;
(ii) A documented history of bisphosphonate administration for osteoporosis or malignant disease;
(iii) Availability of complete medical records, including demographic information, medical history, and clinical findings;
(iv) Diagnosis and follow-up performed at the Oral and Maxillofacial Surgery Department of the Emergency County Clinical Hospital of Craiova between January 2020 and December 2025. Patients meeting these criteria were included in both the statistical and clinical components of the study.

The diagnosis of BRONJ was established according to the diagnostic framework proposed by the American Association of Oral and Maxillofacial Surgeons (AAOMS) in its 2022 Position Paper on medication-related osteonecrosis of the jaw. For the purposes of the present study, eligible patients were required to have a current or previous history of bisphosphonate therapy, exposed bone or bone that could be probed through an intraoral or extraoral fistula in the maxillofacial region persisting for more than eight weeks, and no history of radiation therapy to the jaws or metastatic disease involving the jaws. Since the present investigation was restricted exclusively to patients exposed to bisphosphonates, the term bisphosphonate-related osteonecrosis of the jaw (BRONJ) was used consistently when referring to the study cohort.

### 2.5. Exclusion Criteria

Duplicate records were removed during data cleaning and were not treated as independent patients. After consolidation, patients were excluded from the BRONJ-specific study population if osteonecrosis was not associated with bisphosphonate therapy (*n* = 27), if the available documentation was insufficient to confirm BRONJ according to the predefined diagnostic criteria, or if essential information on bisphosphonate exposure or clinical findings was unavailable. The final analytical cohort consisted exclusively of the 94 eligible patients with bisphosphonate-associated BRONJ.

The study was conducted in accordance with the principles of the Declaration of Helsinki and was approved by the Ethics Committee of the University of Medicine and Pharmacy of Craiova (Approval No. 108/27 January 2025). Patient data were retrospectively retrieved from existing medical records and processed in anonymized form to ensure confidentiality.

## 3. Results

Following removal of duplicate records, 121 unique patients with osteonecrosis of the jaw were identified. Of these, 94 patients met the eligibility criteria for jaw osteonecrosis associated with bisphosphonate therapy and constituted the final analytical sample. All demographic and association analyses in the revised manuscript are to be based exclusively on these 94 patients (Table 1). The previously reported sex, age, residence, diabetes, and hypertension distributions require reconciliation with the final patient-level database before numerical results are reported. Among the 94 eligible BRONJ cases, 75 (79.8%) had received bisphosphonate therapy for malignant disease and 19 (20.2%) for osteoporosis (Table 2). Any interpretation attributing the distribution of urban and rural cases to transport infrastructure or access to care was removed because this hypothesis was not evaluated in the present study.

Regarding anatomical distribution, mandibular involvement was observed in 77.0% of cases, whereas maxillary involvement was recorded in 23.0%, with no cases involving both jaws. Posterior regions predominated in both the mandible and maxilla (Table 3).

The most frequent clinical findings were exposed necrotic bone and a grayish appearance of the exposed bone, both present in all patients. Purulent discharge and oral fistula were observed in 37% and 35% of cases, respectively (Table 4).

The age distribution did not differ significantly according to the presence of malignant tumors, diabetes mellitus, or osteoporosis, and the corresponding effect sizes were very small (Table 5).

The most statistically robust result is the association between gender and osteoporosis, followed by the association between gender and the presence of malignant tumors.

As shown in Table 6, the distribution of AAOMS clinical stages differed according to the primary indication for bisphosphonate therapy.

### Results of Clinical Study

**Figure 3:**
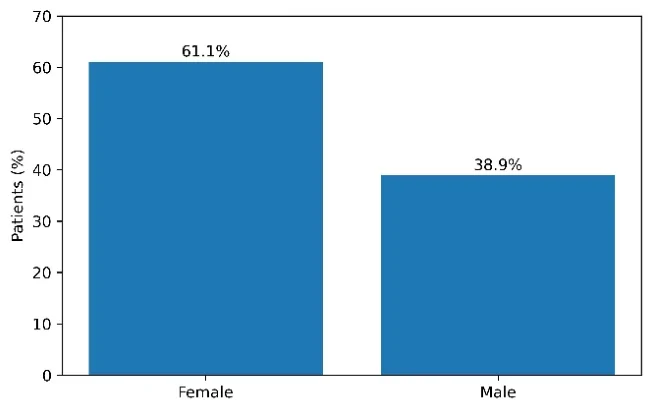
Sex distribution of the subjects.

The clinical observational study comprised 94 patients diagnosed with bisphosphonate-related osteonecrosis of the jaw (BRONJ). Most patients had received antiresorptive treatment for malignant diseases (79.8%), whereas 20.2% had been treated for osteoporosis (Figure 3). This distribution indicates that the clinical cohort was predominantly represented by oncology patients undergoing bisphosphonate therapy.

**Figure 4:**
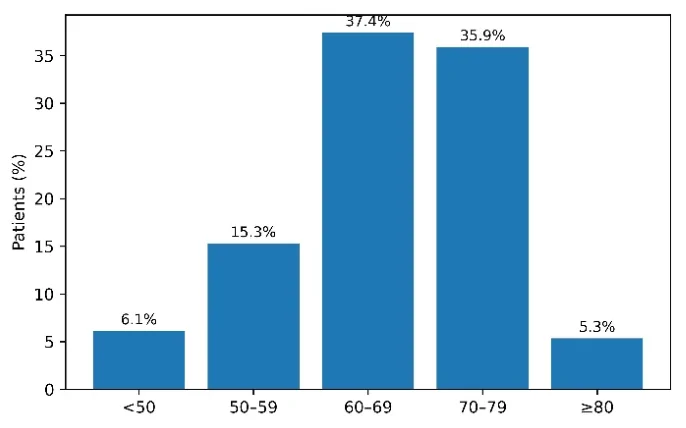
Age groups of the subjects.

Evaluation of lesion distribution demonstrated a marked predominance of mandibular involvement (77%), while 23% of the lesions affected the maxilla (Figure 4). Notably, none of the patients presented simultaneous involvement of both jaws, suggesting that the disease was confined to a single anatomical site in all cases included in the study.

**Figure 5:**
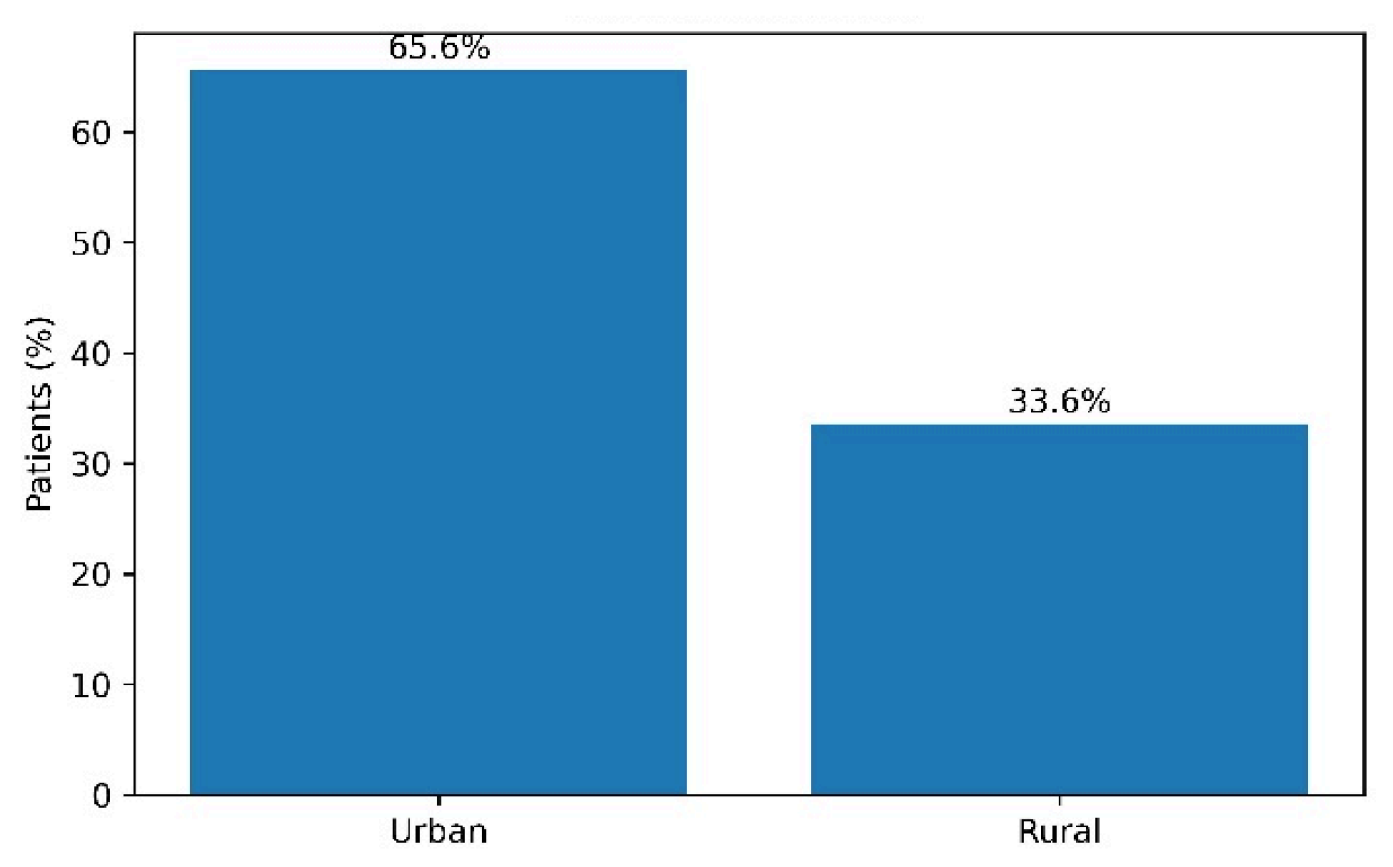
Area of residence of the subjects.

**Figure 6:**
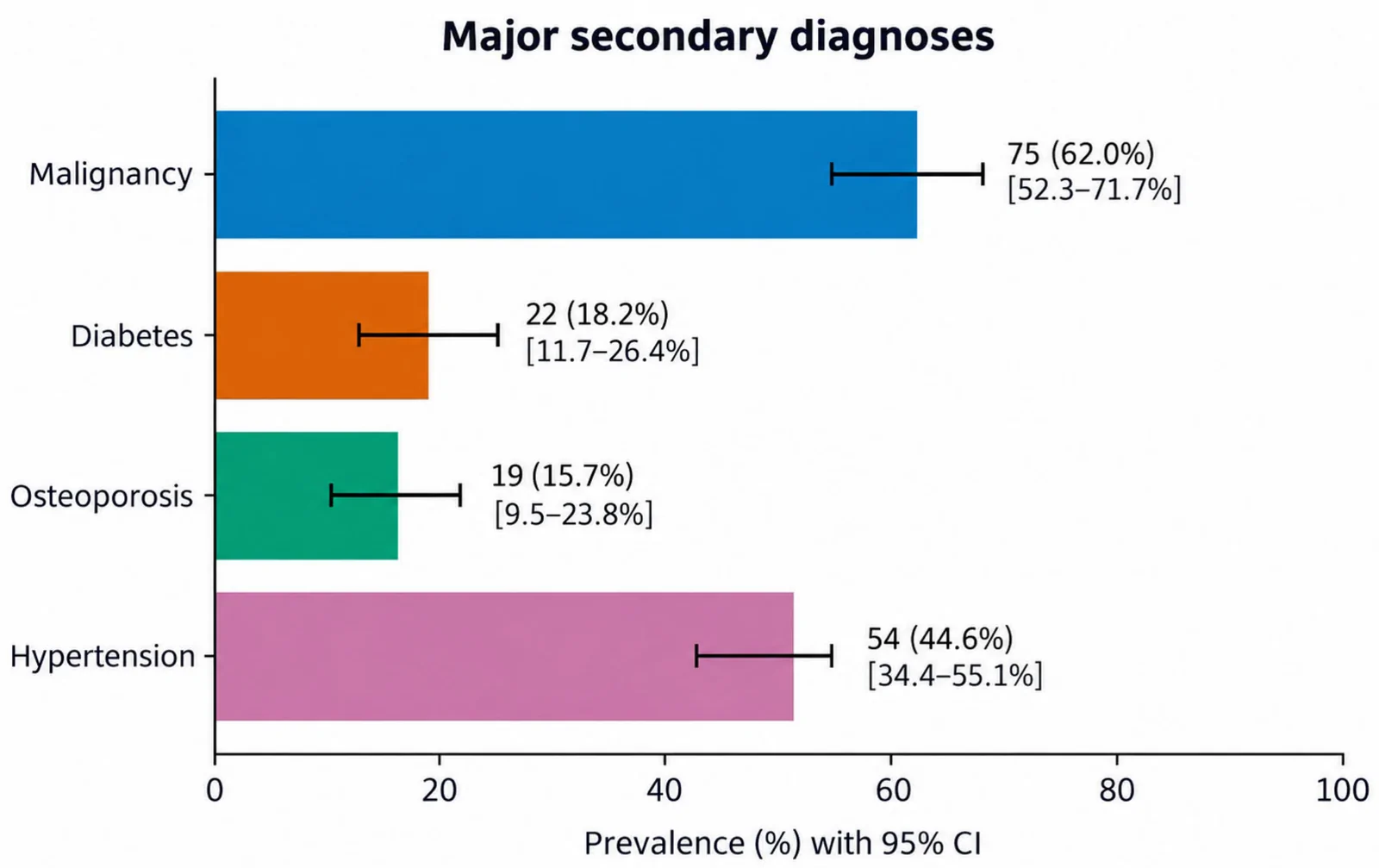
Major secondary diagnoses of the subjects.

Among mandibular lesions, the posterior premolar–molar region represented the most frequently affected site, accounting for 88% of mandibular cases (Figure 5). Similarly, maxillary lesions showed a clear preference for the posterior maxilla and the maxillary tuberosity, which together represented 91% of all maxillary localizations (Figure 6). These findings indicate a consistent tendency for BRONJ to develop within the posterior regions of both jaws.

**Figure 7:**
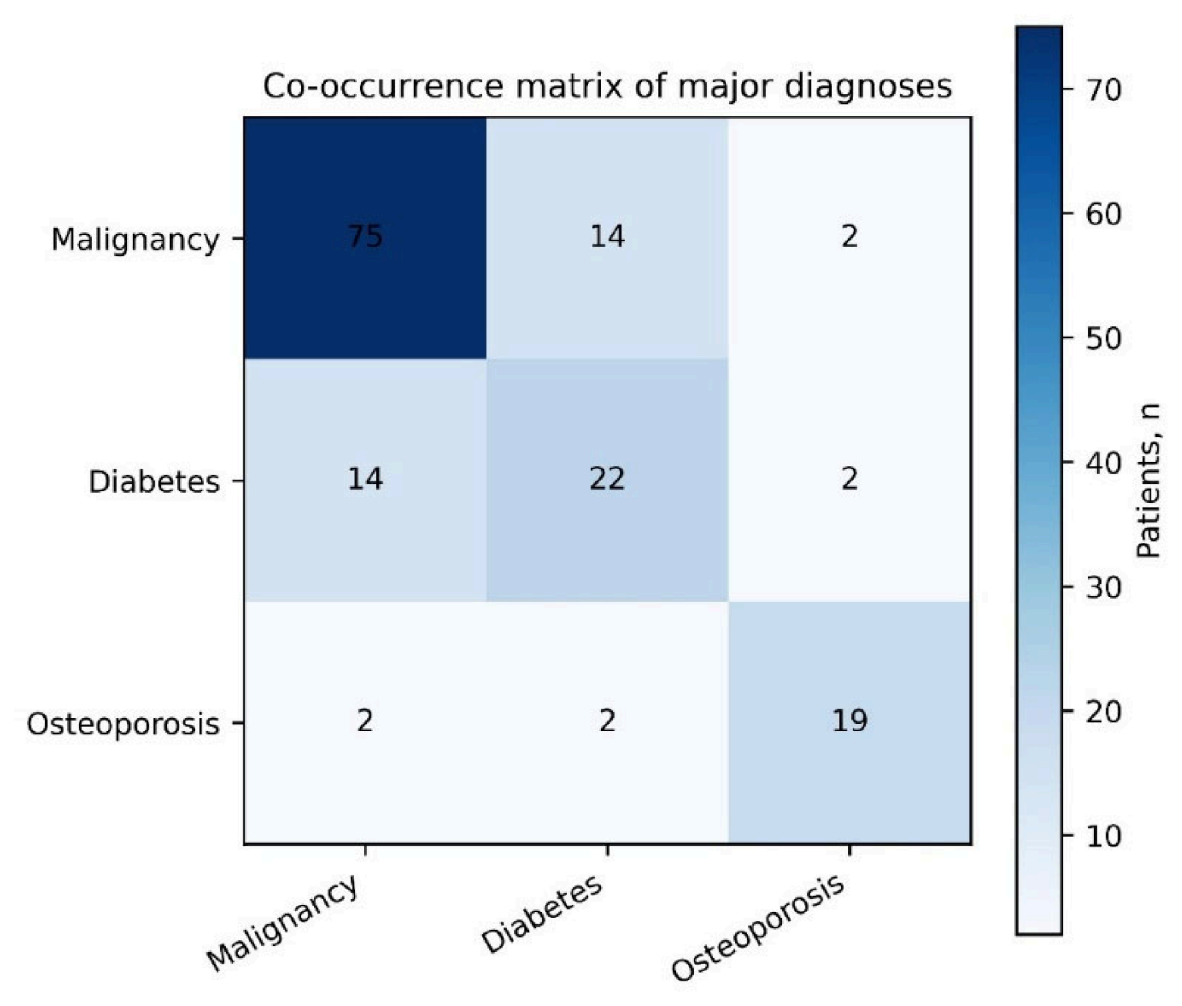
Co-occurrence matrix of major diagnoses of the subjects.

Clinical examination identified exposed necrotic bone and grayish bone discoloration in every patient, confirming these features as the predominant clinical manifestations of BRONJ (Figure 7). In addition, purulent discharge was observed in 37% of patients, while oral fistulas were recorded in 35%, reflecting the frequent association of BRONJ with secondary inflammatory or infectious complications.

**Figure 8:**
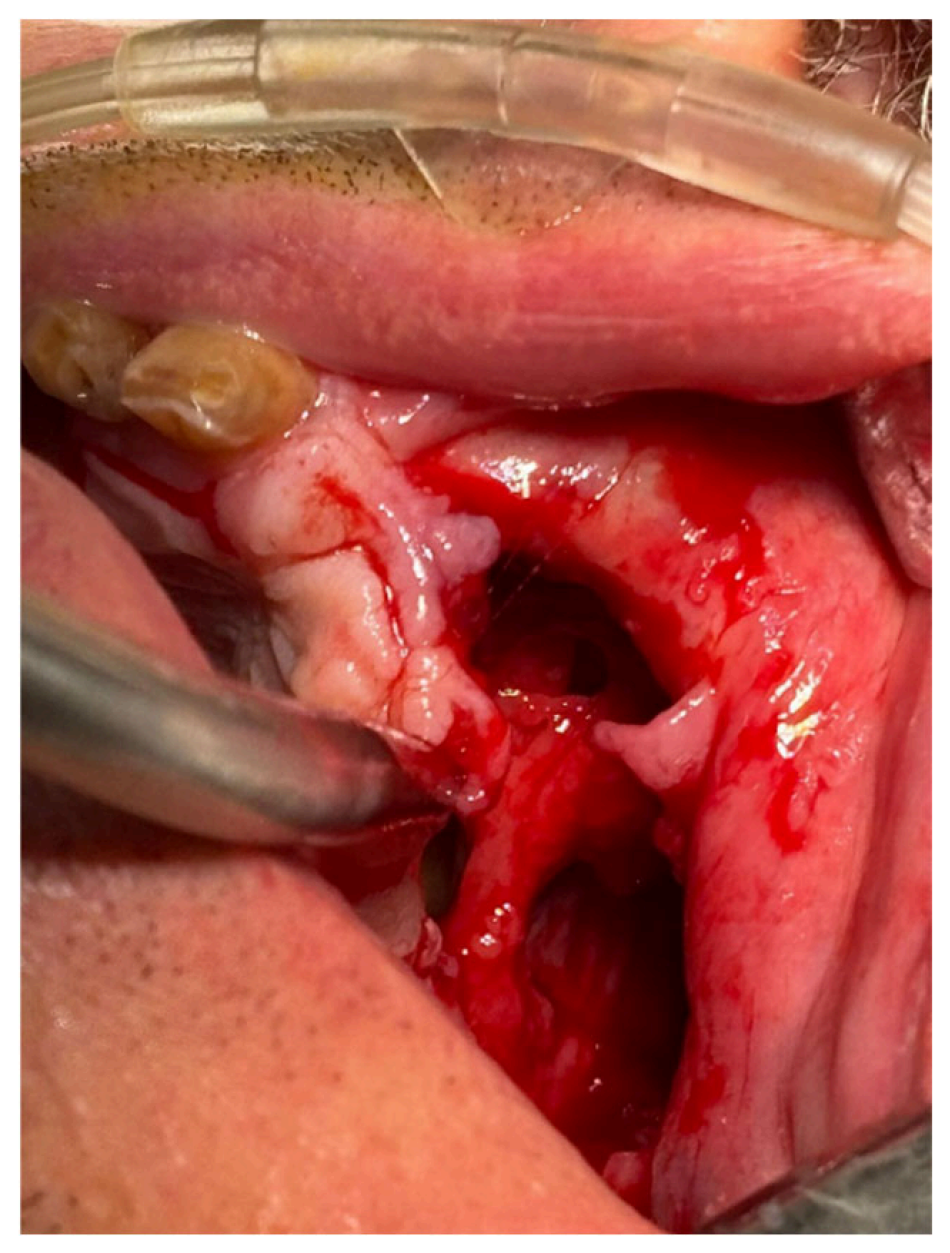
Intraoperative view showing surgical removal of necrotic bone from the maxilla in a patient with bisphosphonate-related osteonecrosis of the jaw (BRONJ).

The clinical symptom profile included localized pain, oral discomfort, hypoesthesia, paresthesia, pain during mastication, and swallowing difficulties (Figure 8). Collectively, these manifestations illustrate the heterogeneous clinical presentation of BRONJ and emphasize the functional impairment associated with this condition.

Figure 8 illustrates the clinical assessment workflow used for patients with BRONJ. The evaluation incorporated medical and medication history, previous dentoalveolar procedures, comprehensive intraoral examination, anatomical localization of the lesion, clinical signs, and patient-reported symptoms. This structured approach was used to organize the clinical information available in the medical records and to characterize the presentation of BRONJ within the study sample.

**Figure 9:**
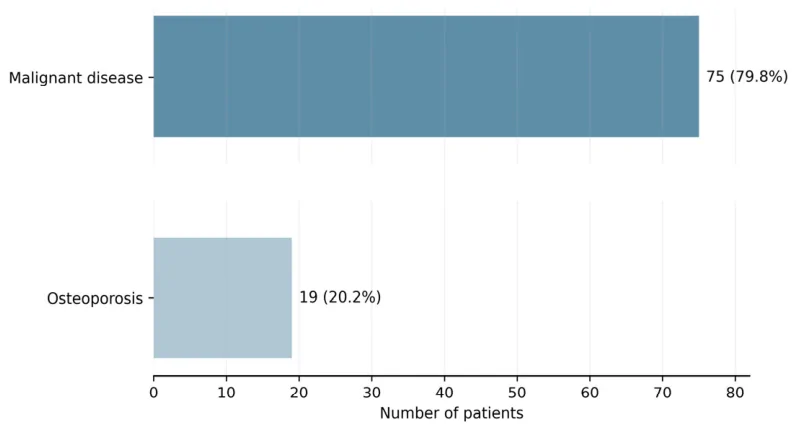
Primary indication for bisphosphonate therapy (*n* = 94).

Among the 94 eligible BRONJ cases, 75 (79.8%) had received bisphosphonate therapy for malignant disease and 19 (20.2%) for osteoporosis. The distribution according to the primary indication for bisphosphonate therapy is illustrated in Figure 9.

**Figure 10:**
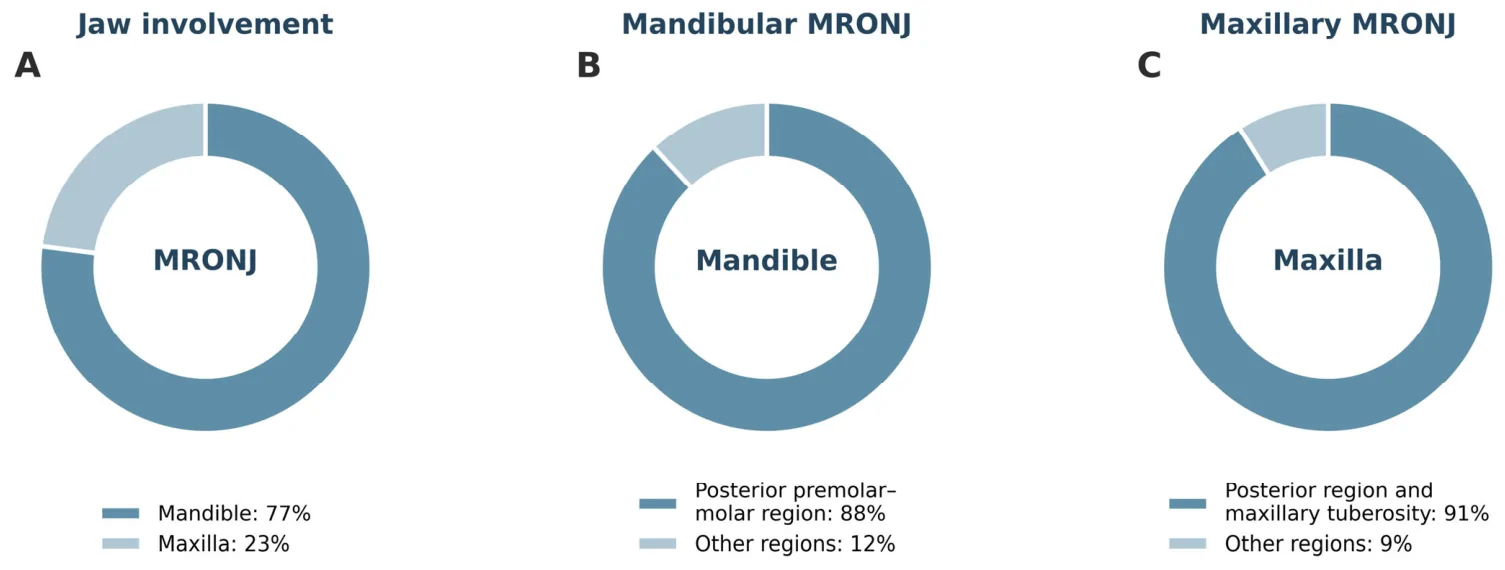
Anatomical distribution of bisphosphonate-related osteonecrosis of the jaw (BRONJ) lesions in the study population. (**A**) Distribution according to jaw involvement, showing mandibular involvement in 77% and maxillary involvement in 23% of cases. (**B**) Topographic distribution of mandibular BRONJ, with 88% of lesions involving the posterior premolar–molar region. (**C**) Topographic distribution of maxillary BRONJ, with 91% of lesions involving the posterior region and maxillary tuberosity. No simultaneous involvement of both jaws was identified.

Regarding anatomical distribution, mandibular involvement was observed in 77% of cases and maxillary involvement in 23%, with no simultaneous involvement of both jaws. Posterior regions predominated in both anatomical locations, as illustrated in Figure 10.

**Figure 11:**
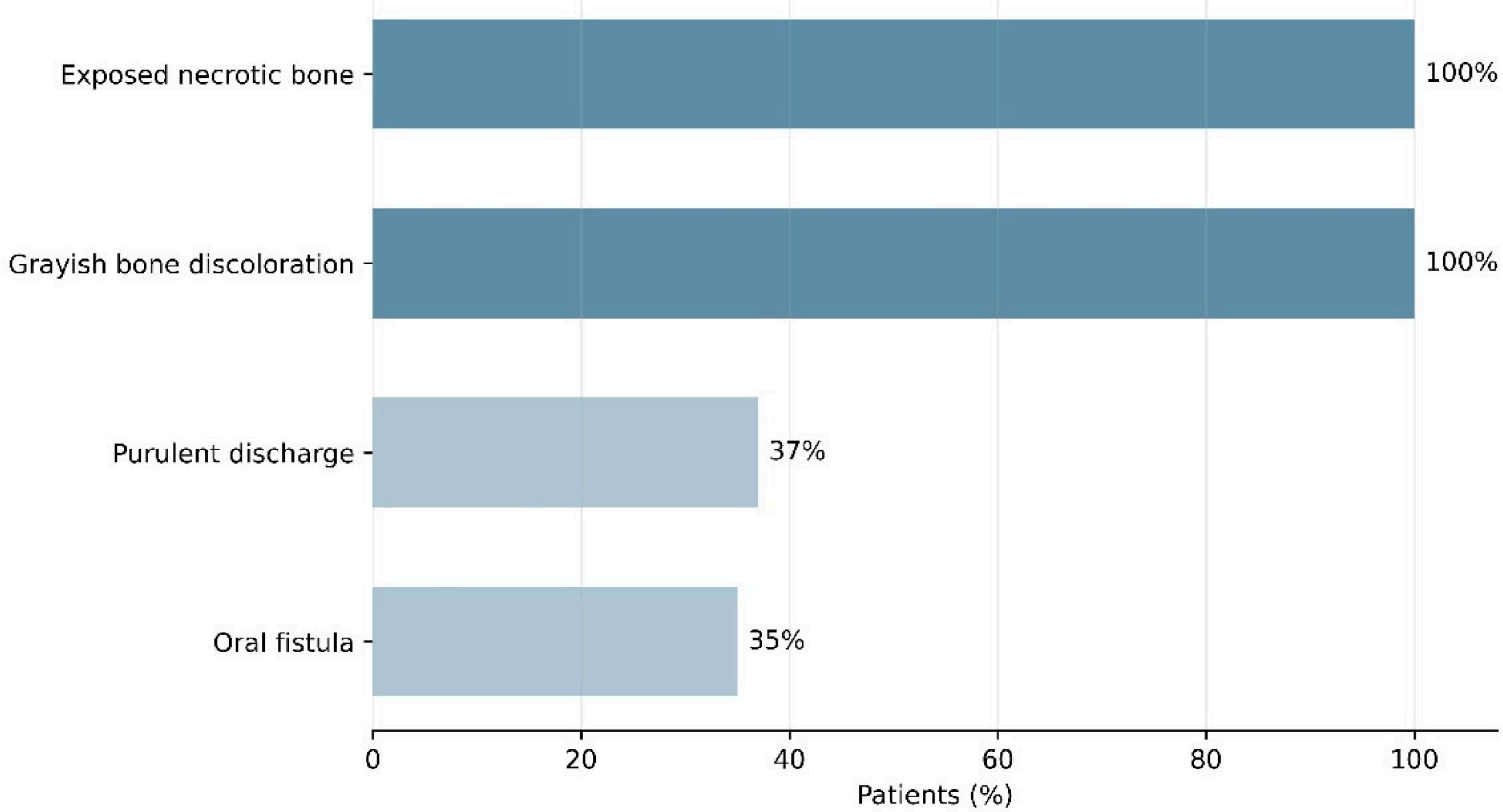
Topographic distribution of mandibular (BRONJ).

A more detailed representation of the topographic distribution of mandibular BRONJ lesions is presented in Figure 11. The principal clinical signs identified during intraoral examination included exposed necrotic bone, grayish discoloration of the exposed bone, purulent discharge, and oral fistula.

**Figure 12:**
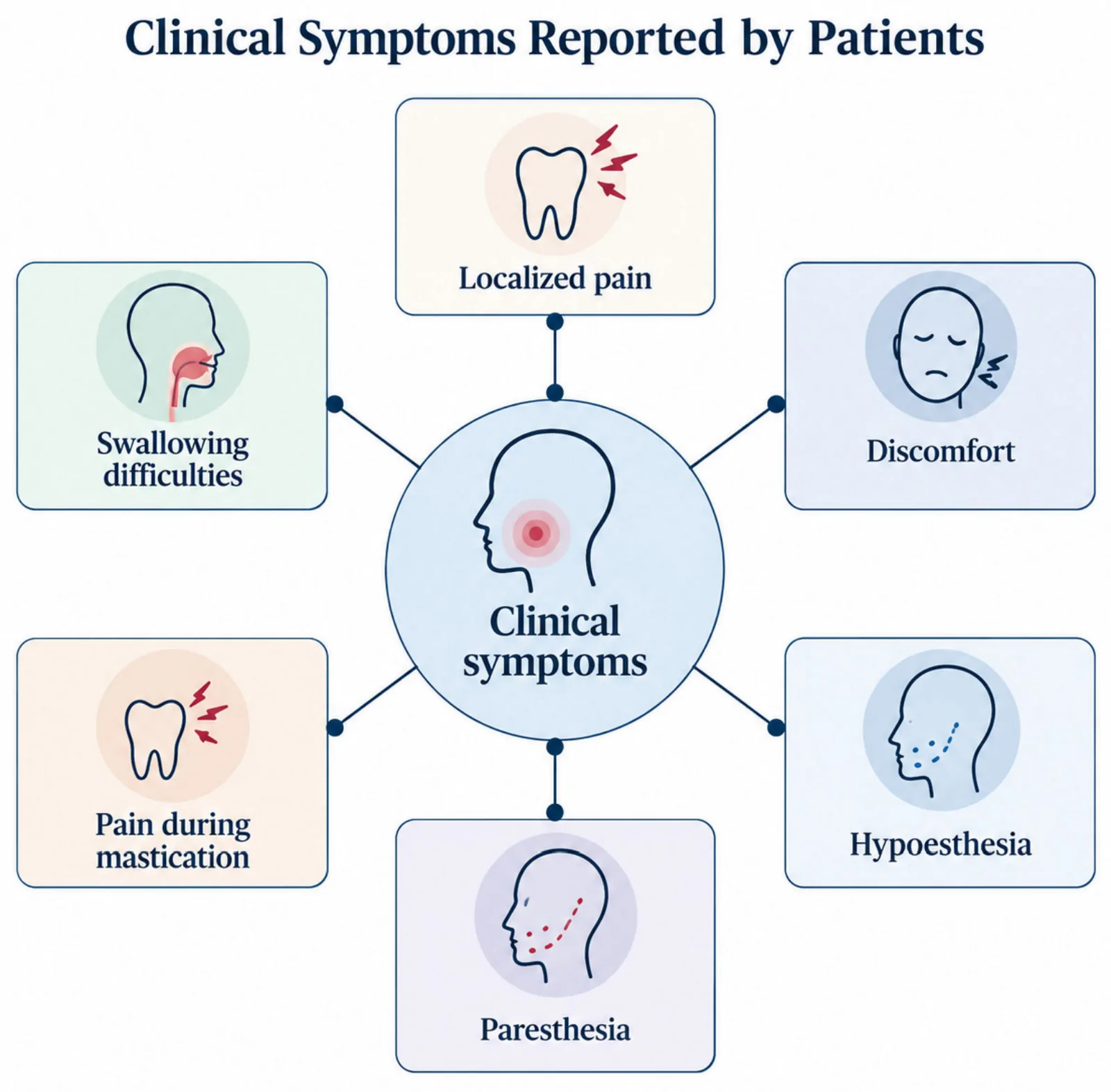
Main clinical signs observed during intraoral examination.

The clinical symptom profile included localized pain, oral discomfort, hypoesthesia, paresthesia, pain during mastication, and swallowing difficulties (Figure 12).

**Figure 13:**
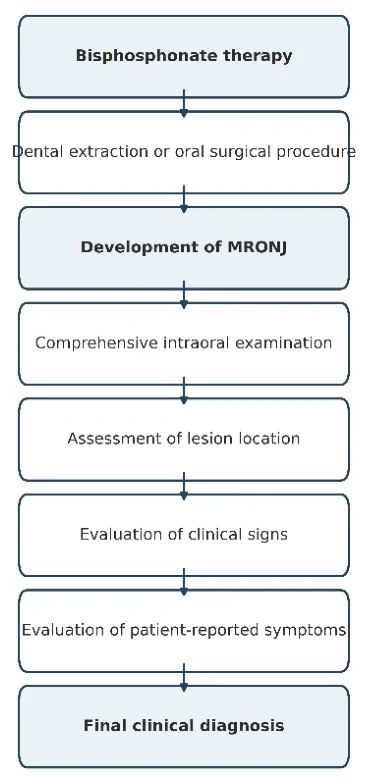
Clinical diagnostic workflow.

The diagnostic approach followed for the clinical evaluation of patients with BRONJ is presented in Figure 13.

## 4. Discussion

The present study focused on 94 patients with BRONJ who were identified from 121 unique patients with osteonecrosis of the jaw after database cleaning. Among the 94 BRONJ cases, 75 (79.8%) had received bisphosphonate treatment for malignant disease and 19 (20.2%) for osteoporosis. This distribution describes the case mix observed at our referral center and should not be interpreted as evidence that one treatment indication or administration route confers a higher BRONJ risk than another. Such a comparison would require the total number of bisphosphonate-exposed patients in each treatment group, including those who did not develop BRONJ, which was not available in the present dataset. The drug and route information should therefore be presented descriptively and only to the extent supported by the original patient-level records.

The anatomical distribution—mandibular involvement in 77% of cases versus 23% maxillary, with no bilateral cases—matches previously reported ratios of roughly 58–77% mandibular predominance, attributed to the mandible’s comparatively lower vascularity and denser cortical bone [13,14,15]. The concentration of lesions in the posterior premolar–molar region of the mandible (88%) and the posterior maxilla/tuberosity (91%) is likewise consistent with a reported trend toward posterior predominance in dentoalveolar-surgery-associated BRONJ [16]. The marked mandibular predominance observed in our series may be interpreted in the context of the distinctive biological and anatomical characteristics of the jaws. Jawbone is characterized by continuous remodeling driven by mastication, periodontal loading, and repeated microtrauma, while dentoalveolar procedures may further increase local remodeling requirements. Suppression of osteoclast-mediated bone turnover by bisphosphonates may therefore impair the ability of the affected bone to respond adequately to local injury. The posterior mandible may be particularly susceptible because of its relatively dense cortical architecture and comparatively limited vascular supply compared with the maxilla. Furthermore, the close relationship between alveolar bone, periodontal tissues, and the oral microbiota provides a potential pathway for local inflammatory and infectious processes to contribute to the persistence and progression of exposed necrotic bone. These anatomical and biological characteristics may partly explain the preferential mandibular distribution observed in the present cohort, although the retrospective design of our study does not allow direct assessment of these mechanisms. The frequency of oral fistulas (35%) and purulent secretions (37%) in our sample also falls within the range reported elsewhere, where suppuration and fistula formation are common, though not universal, features of exposed necrotic bone [17,18]—together with the pain, hypoesthesia/paresthesia, and chewing/swallowing difficulties reported here, underscoring the substantial functional burden of BRONJ. In this context, the differential diagnosis of suspected BRONJ remains clinically important, because several inflammatory, odontogenic, osseous, sinonasal, and malignant conditions may produce overlapping clinical or radiological findings [19,20,21,22,23,24,25,26,27,28,29,30,31]. Exposed necrotic bone is a characteristic clinical feature of BRONJ. However, in patients without visible bone exposure, the differential diagnosis should include periodontal and periapical disease, sinusitis, gingivitis or mucositis, temporomandibular disorders, osteomyelitis, metastatic bone lesions, neuralgia-associated osteonecrosis, and osteoradionecrosis. Additionally, several conditions may also present with exposed bone despite being unrelated to bisphosphonate therapy. These include cemento-osseous dysplasia with secondary sequestration, traumatic bone exposure, infectious osteomyelitis, osteonecrosis following herpes zoster infection, and HIV-associated necrotizing ulcerative periodontitis.

When considered alongside data from other geographical settings, our findings show both similarities and differences in the clinical profile of BRONJ. In our series, 75 of the 94 patients (79.8%) had received bisphosphonate therapy for malignant disease, whereas 19 patients (20.2%) had been treated for osteoporosis. This distribution can be compared with a recent large European multicenter investigation involving 537 BRONJ patients, in which metastatic bone disease also represented an important indication for antiresorptive treatment [32]. Data from a Central European multicenter population likewise suggest that the underlying systemic condition and the characteristics of antiresorptive exposure contribute substantially to the heterogeneity observed among BRONJ case series [33]. Nevertheless, differences in patient referral, therapeutic indications, and treatment protocols between centers make direct numerical comparisons difficult.

The anatomical pattern identified in our cohort also shows considerable agreement with observations from other populations. Mandibular involvement accounted for 77% of our cases, and 88% of mandibular lesions were located in the posterior premolar–molar region. A nationwide Japanese survey similarly reported substantially more mandibular than maxillary cases, although simultaneous involvement of both jaws was also documented in a minority of patients [34]. In contrast, none of the patients in our cohort presented simultaneous maxillary and mandibular involvement. Stage II was the predominant clinical presentation in our study, accounting for 74.5% of cases, a pattern that has also been frequently reported in other BRONJ populations [33,34]. The association between BRONJ stage and underlying disease should be interpreted as pharmacologically expected rather than as a novel finding, given the differences in antiresorptive exposure between malignancy and osteoporosis. Recent nationwide Korean data further illustrate that the epidemiological profile of BRONJ may vary according to patient characteristics and patterns of antiresorptive exposure [35]. Taken together, these international comparisons suggest that the mandibular predominance and frequent Stage II presentation observed in our Romanian cohort are consistent with broader BRONJ experience, whereas the high proportion of patients treated for malignant disease may partly reflect the characteristics of the population referred to our oral and maxillofacial surgery center. These findings should therefore be interpreted as characteristics of the BRONJ cases managed at our institution rather than as evidence of a higher population-level risk associated with a particular systemic disease or bisphosphonate regimen.

Associations between sex, age, residence, and systemic diagnoses should be interpreted only after recalculation using the final 94-patient cohort. The analyses originally performed on 121 unique osteonecrosis patients cannot be used to draw BRONJ-specific conclusions because they included 27 patients whose osteonecrosis was unrelated to bisphosphonate therapy. In the revised analysis, effect estimates and *p* values will therefore be reported exclusively for the eligible BRONJ cases. Any interpretation of sex-related differences will be framed as an association within this institutional case series rather than as evidence of sex-specific susceptibility to BRONJ.

The retrospective dataset was derived from medical records collected over a six-year period and required consolidation of repeated entries before identification of the final BRONJ sample. This data-cleaning step reduced duplication but cannot completely eliminate the possibility of residual misclassification or incomplete documentation. The analyses were exploratory, and no adjustment for multiple comparisons was planned; consequently, any statistically significant associations should be regarded as hypothesis-generating. Furthermore, the single-center design and the absence of a non-BRONJ comparison population limit generalizability and preclude estimation of incidence or causal treatment effects.

Overall, the findings characterize the clinical pattern of BRONJ cases treated at our center, including the predominance of malignant disease as the treatment indication and the frequent involvement of posterior jaw regions. Because the study contains only identified BRONJ cases and lacks denominators for the total populations exposed to oral and intravenous bisphosphonate regimens, no direct comparison of regimen-specific risk can be made. The results nevertheless support careful dental evaluation before antiresorptive therapy and continued oral-health surveillance during treatment, particularly in medically complex patients.

### 4.1. Study Limitations

The retrospective nature of the study imposed dependence on the completeness and accuracy of existing medical records, and some treatment-related variables were not consistently documented.

The study was conducted at a single tertiary referral center; therefore, referral patterns and local prescribing practices may limit the external generalizability of the findings.

The final sample size was determined by the number of eligible cases available during the study period, and several subgroup analyses contained small cell counts, reducing statistical precision and power. In addition, detailed information on bisphosphonate dose, cumulative exposure, treatment duration, and route of administration was not consistently available at the individual-patient level. Most importantly, the study did not include a control group or the total number of patients exposed to oral or intravenous bisphosphonates who did not develop BRONJ. Consequently, incidence, relative or absolute risk, and causal relationships between treatment regimen and BRONJ cannot be estimated from these data. Comparisons between drug regimens must therefore be interpreted strictly as distributions among identified BRONJ cases.

### 4.2. Study Strengths

Despite these limitations, the study has several important strengths. It combines a comprehensive statistical evaluation with a detailed clinical assessment performed on the same cohort of patients with BRONJ. The inclusion of patients diagnosed over a six-year period using standardized clinical criteria enhances the consistency of the findings, while the integration of demographic, clinical, and anatomical characteristics provides a comprehensive overview of BRONJ presentation in routine clinical practice. Furthermore, the rigorous data-cleaning procedure minimized duplicate records and improved the reliability of the statistical analyses.

Future perspectives: Multicenter prospective studies including radiological staging, treatment outcomes and long-term follow-up are warranted.

## 5. Conclusions

In conclusion, among the bisphosphonate-related osteonecrosis of the jaw (BRONJ) cases identified at this single referral center, most patients had received treatment for malignant disease, and lesions most frequently involved the mandible, particularly posterior sites. These findings describe the clinical and anatomical profile of the study population but do not establish comparative treatment risk or causality. The observed distribution of clinical stage and underlying systemic disease warrants further evaluation in larger, multicenter studies with complete treatment-exposure data and appropriate comparison groups. From a clinical perspective, the findings support dental assessment before bisphosphonate initiation and coordinated multidisciplinary follow-up during therapy.

## Figures and Tables

**Table 1:** Distribution of bisphosphonate therapy in the final BRONJ cohort.

Bisphosphonate	*n*	%
Alendronate	69	73.0
Zoledronate	25	27.0

**Table 2:** Primary indication for bisphosphonate therapy in the final BRONJ cohort.

Primary Indication for Bisphosphonate Therapy	*n*	%
Malignant disease	75	79.8
Osteoporosis	19	20.2
Total	94	100.0

Table legend: Distribution of patients according to the primary indication for bisphosphonate therapy. Patients presenting both malignant disease and osteoporosis were classified according to the primary indication for which bisphosphonate therapy had been prescribed. In patients presenting both conditions, bisphosphonate treatment was administered for malignant disease.

**Table 3:** Anatomical distribution of BRONJ.

Anatomical Characteristic	%
Mandible	77.0
Maxilla	23.0
Both jaws	0.0
Posterior premolar–molar region among mandibular cases	88.0
Posterior region/maxillary tuberosity among maxillary cases	91.0

Table legend: Anatomical distribution of BRONJ lesions within the final clinical cohort (*n* = 94). Percentages describing mandibular and maxillary sublocations refer to patients affected in the respective jaw.

**Table 4:** Clinical signs observed in patients with BRONJ.

Clinical Finding	%
Exposed necrotic bone	100
Grayish appearance of exposed bone	100
Purulent discharge	37
Oral fistula	35

Table legend: Principal clinical findings recorded during intraoral examination of patients with bisphosphonate-associated osteonecrosis of the jaw.

**Table 5:** Comparison of age according to major secondary diagnoses.

Outcome	Present, *n*	Age Present, Median (IQR)	Absent, *n*	Age Absent, Median (IQR)	Mann–Whitney U	*p*	Rank-Biserial r
Malignant tumors	75	66 (61–74)	46	68.5 (63.25–73.5)	1593.5	0.4839	−0.076
Diabetes mellitus	19	68 (62–71.5)	102	67 (61–74)	964.5	0.9772	−0.005
Osteoporosis	19	68 (61–70)	102	67 (61.25–74)	874.0	0.5005	−0.098

The age distribution did not differ significantly based on the presence of malignant tumors, diabetes, or osteoporosis. The effect sizes were very small. **Calculation method:** The age distribution differed significantly from a normal distribution (Shapiro–Wilk *p* < 0.001). Therefore, comparisons were performed using the two-sided Mann–Whitney U test. Rank–biserial correlation was reported as the effect size.

**Table 6:** Association between AAOMS clinical stage and the primary indication for bisphosphonate therapy.

AAOMS Stage	Osteoporosis, *n*/*N* (%)	Malignant Disease, *n*/*N* (%)	Total, *n* (%)
Stage I	13/19 (68.4)	0/75 (0.0)	13 (13.8)
Stage II	6/19 (31.6)	64/75 (85.3)	70 (74.5)
Stage III	0/19 (0.0)	11/75 (14.7)	11 (11.7)
**Total**	**19 (100)**	**75 (100)**	**94 (100)**

A significant association was identified between the clinical stage of BRONJ and the primary indication for bisphosphonate therapy (χ^2^ = 59.98, *p* < 0.001; Cramér’s V = 0.799). Stage I disease was observed exclusively among patients treated for osteoporosis, whereas all Stage III cases occurred among patients receiving bisphosphonates for malignant disease. Stage II represented the most frequent presentation overall and accounted for 74.5% of the final BRONJ cohort. These findings describe differences in clinical presentation within the study sample and should not be interpreted as estimates of treatment-related risk.

## Data Availability

The data supporting the findings of this study are available from the corresponding author upon reasonable request. The data are not publicly available due to privacy and ethical considerations related to the protection of participants’ personal information.
